# The Role of Melatonin as a Hormone and an Antioxidant in the Control of Fish Reproduction

**DOI:** 10.3389/fendo.2016.00038

**Published:** 2016-05-04

**Authors:** Saumen Kumar Maitra, Kazi Nurul Hasan

**Affiliations:** ^1^Department of Zoology, Visva-Bharati University, Santiniketan, India

**Keywords:** antioxidant, fish, hormone, melatonin, reproduction

## Abstract

Reproduction in most fish is seasonal or periodic, and the spawning occurs in an appropriate season to ensure maximum survival of the offspring. The sequence of reproductive events in an annual cycle is largely under the control of a species-specific endogenous timing system, which essentially relies on a well-equipped physiological response mechanism to changing environmental cues. The duration of solar light or photoperiod is one of the most predictable environmental signals used by a large number of animals including fish to coordinate their seasonal breeding. In vertebrates, the pineal gland is the major photoneuroendocrine part of the brain that rhythmically synthesizes and releases melatonin (*N*-acetyl-5-methoxytryptamine) into the circulation in synchronization with the environmental light–dark cycle. Past few decades witnessed an enormous progress in understanding the mechanisms by which melatonin regulates seasonal reproduction in fish and in other vertebrates. Most studies emphasized hormonal actions of melatonin through its high-affinity, pertussis toxin-sensitive G-protein (guanine nucleotide-binding protein)-coupled receptors on the hypothalamus–pituitary–gonad (HPG) axis of fish. However, the discovery that melatonin due to its lipophilic nature can easily cross the plasma membrane of all cells and may act as a potent scavenger of free radicals and stimulant of different antioxidants added a new dimension to the idea explaining mechanisms of melatonin actions in the regulation of ovarian functions. The basic concept on the actions of melatonin as an antioxidant emerged from mammalian studies. Recently, however, some new studies clearly suggested that melatonin, apart from playing the role of a hormone, may also be associated with the reduction in oxidative stress to augment ovarian functions during spawning. This review thus aims to bring together the current knowledge on the role of melatonin as a hormone as well as an antioxidant in the control of fish reproduction and shape the current working hypotheses supported by recent findings obtained in carp or based on knowledge gathered in mammalian and avian species. In essence, this review highlights potential actions of melatonin as a hormone in determining temporal pattern of spawning and as an antioxidant in regulating oocyte maturation at the downstream of HPG axis in fish.

## Introduction

Most fish are discontinuous or seasonal breeders. They exhibit peak reproductive activity or spawning for a short period followed by a long and complicated cascade mechanism of preparation. In an annual cycle, periodic reproductive events often synchronize with seasonal changes in one or other of a group of environmental cues to ensure that breeding occurs in the utmost favorable part of the year. Organisms have the ability to measure the time by their biological rhythms. The environmental components, which constitute the informative reservoir used by a fish species in timing seasonal reproduction, broadly include solar day length or photoperiod, temperature, rainfall, nutrients, and food supply. On the other hand, some limnological features, such as pH, turbidity, salinity, dissolved oxygen, and total alkalinity, and other factors, such as lunar periodicity, may determine the final act of spawning (1).

During the course of evolution, animals in general have developed different responsive mechanisms to the stimuli associated with seasonal changes in the environment to adequately adapt to its own physiological changes and energy demands. However, seasonal changes in day length individually, or in combination with temperature, lend an imperative contribution to the mechanisms that synchronize seasonal reproduction and eventually affect the “driving function” in reorienting the breeding periodicity in most temperate fish species (2). The external cues synchronize and modulate the circadian clock, which in turn determines rhythmic production of messengers that affect the target cells and tissues to control different body functions. Physiologically, concentrations and activity of a variety of hormones and neurotransmitter-like substances govern the circadian and circannual rhythms, which ultimately help the animals to their respective adaptive functions. In different vertebrates, sensors and circadian oscillators, such as the pineal organ, lateral eyes, and suprachiasmatic nuclei (SCN) of hypothalamus, built up a system for synchronization of seasonal reproduction with the environment (3). This circadian oscillator system among the fish species is located in the pineal organ and the eyes (4), both of which are capable of synthesizing and secreting melatonin (*N*-acetyl-5-methoxytryptamine) in rhythmic fashion (5). The fish pineal organ, due to its unique property of direct responsiveness to the changes in light–dark conditions of environment (6), is considered as the most important component of the neuroendocrine system. Melatonin employs to measure and predict daily and seasonal time in determining periodic reproductive events in an annual cycle (7–10).

In fish, as in other vertebrates, melatonin acts as a conservative chemical messenger of photoperiod or *Zeitgeber*, and plasma melatonin titers remain high during the dark phase and low during daytime (11, 12). In recognizing this characteristic feature, melatonin is also termed as “signal of darkness” or the “time-keeping hormone” of the body (13). The mechanism of photoperiodic and/or circadian control of melatonin synthesis in the pineal gland has greatly varied during evolution, but the melatonin signal released into the blood is the same from fish to mammals (4). Melatonin is a small lipophilic molecule derived from the amino acid tryptophan. Dietary uptake of tryptophan from the circulation into the pineal gland is the prerequisite of melatonin synthesis by five enzymatic actions, of which arylalkylamine *N*-acetyltransferase (AANAT) is the penultimate and rate-limiting enzyme in this biosynthetic pathway (14). Expression of AANAT gene in the pinealocytes of pineal organ employs β-adrenergic system in the mechanism of response to the changes in environmental light–dark conditions in the synthesis and release of melatonin into the circulation (15, 16) to perform its ultimate hormonal actions on the target cells/tissues/organs.

A large number of studies emphasized the role of melatonin as a potent photoneuroendocrine signal of pineal organ in the regulation of reproduction in a wide variety of temperate fish species and in several air-breathing fish (17), but reports on a particular fish species are fragmentary. Thus, a non-air-breathing subtropical carp (*Catla catla*) has been extensively used to gather basic information on the structure and functions of the pineal organ (18) and the role of its hormone melatonin in the regulation of seasonal reproduction (1, 17). This carp species due to its surface dwelling habit maintains close proximity to environmental lighting conditions and thereby offers as an excellent fish model for understanding the photoperiodic control mechanisms of reproductive events in an annual cycle. In recent years, a series of meaningful investigative schedules followed with this fish addressed several important and hitherto neglected issues relating to actions of melatonin on the reproductive functions in fish. As an obvious outcome, melatonin seems to play the role of a hormone in determining the periodicity of spawn and of an antioxidant in controlling final oocyte maturation in an annual cycle.

## Actions of Melatonin as a Hormone in the Regulation of Fish Reproduction

The hormonal role of melatonin in the regulation of fish reproduction is evident from the data gathered from several studies under natural and varied experimental conditions. The findings on temporal pattern of diurnal and seasonal profiles of serum melatonin in relation to the reproductive status of the fish under natural conditions provided the basis of experimental studies, which dealt with dose- and duration-dependent effects of exogenous melatonin on the functions of gonads in different parts of annual reproductive cycle. Additional evidences of hormonal actions of melatonin on fish oocyte maturation emerged from *in vitro* studies and demonstration of specific melatonin receptor proteins on the hypothalamus–pituitary–gonad (HPG) axis of fish as well.

### Diurnal and Seasonal Profiles of Serum Melatonin in Relation to Sexual Status of Fish

Existing knowledge on the rhythm pattern of circulating melatonin in fish is limited mostly to studies on temperate species, in which melatonin levels attain daily peak during the dark phase and fall to basal during the day (4). Seasonal changes in the daily profiles of plasma melatonin in the Atlantic salmon (19) indicate its possible role in the control of periodic reproduction. A shift in the daily rhythm pattern of melatonin in an annual cycle generally attributes to the seasonally changing pattern of day length (7, 20).

The daily pattern of melatonin rhythms is conserved across all vertebrates. However, three variants of nocturnal melatonin profiles, namely, type-A, -B, and -C profiles, are found in different vertebrates, including the fish (2, 4). A-type profiles are characterized by a discrete peak in late dark phase and are found in Atlantic cod (*Gadus morhua*) (20) and haddock (*Melanogrammus aeglefinus*) (21), whereas B-type profiles with a discrete peak in the mid-dark phase are noted in Nile tilapia (*Oreochromis niloticus niloticus*) (22), and the C-type profiles characterizing a rapid rise in melatonin immediately followed by the onset of dark period are found in Atlantic salmon (*Salmo salar*), rainbow trout (*Onchorhynchus mykiss*), Atlantic halibut (*Hippoglossus hippoglossus*), and many other teleosts (2, 4). Few studies also underline environmental regulation of daily and annual melatonin variations and self-sustained endogenous rhythms (2). However, the study on carp reveals for the first time that the nocturnal peak pattern of serum melatonin in a particular fish species may vary from A-type (in the preparatory phase) to B-type (in the remaining parts of annual cycle) in relation to sexual status of fish (23, 24). Since natural day length during preparatory (February–March) phase is not very different from post-spawning (September–October) phase, a shift of daily melatonin peak from one season to the other may be due to a varied relation between the pineal organ and reproduction (19), rather than a response to the length of night (20). Likewise, the peak values of melatonin in different reproductive seasons, as maximum during post-spawning phase and minimum during spawning phase, argue in favor of functional interplay between melatonin levels and oocyte maturation in an annual cycle (25).

### Dose- and Duration-Dependent Effects of Exogenous Melatonin on the Gametogenic and Steroidogenic Functions of Gonads in Different Parts of Annual Breeding Cycle

**Figure 1:**
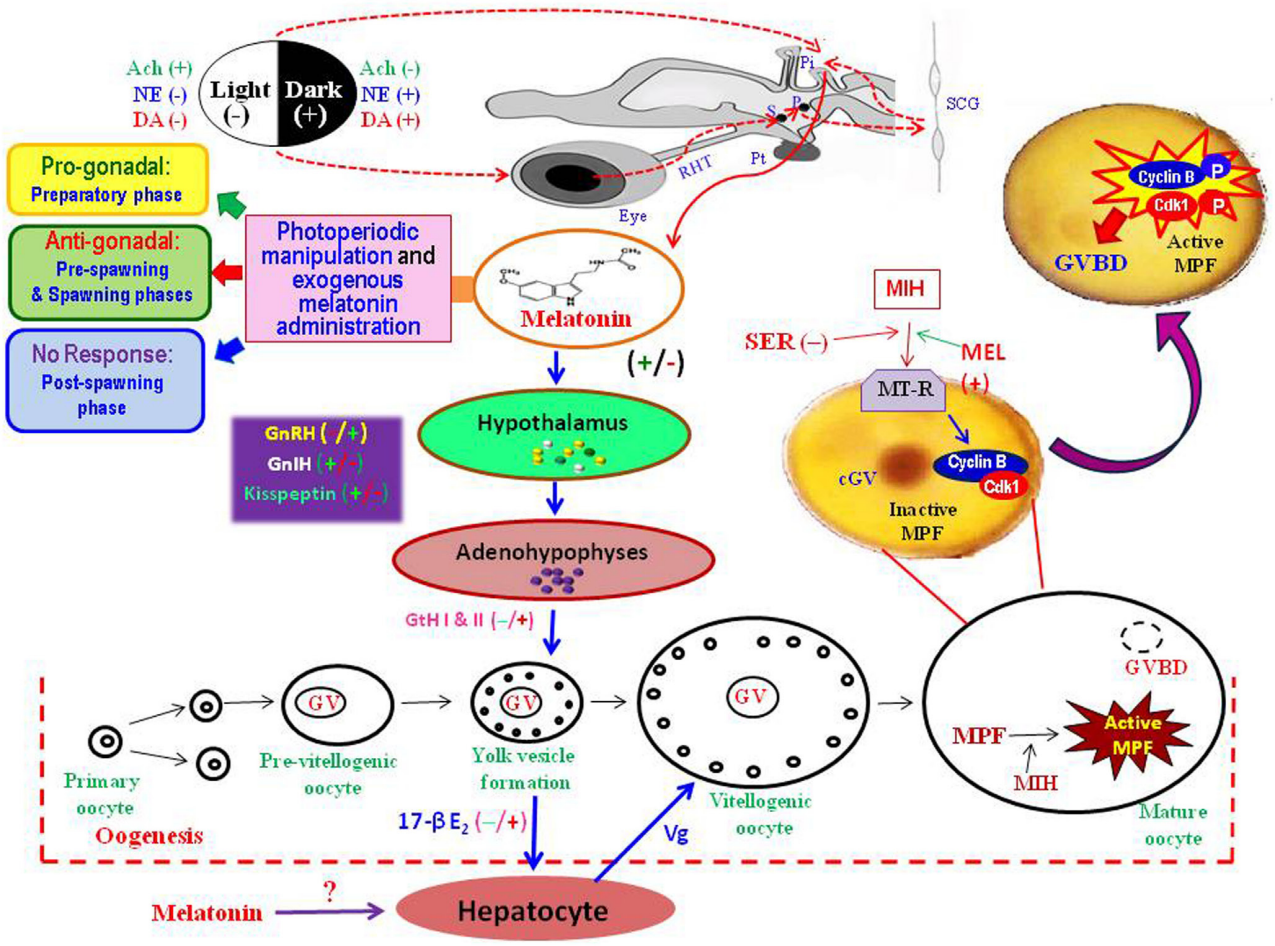
**Diagrammatic presentation of the information gathered from the studies on different fish species to explain possible role of melatonin in the regulation of seasonal events in the ovary**. Photoperiodic stimuli perceived through eye, transmitted through the retino-hypothalamic tract (RHT), *via* a complex pathway involving suprachaismatic nuclei (S), paraventricular nuclei (P), superior cervical ganglion (SCG), and the pineal gland (Pi). The fish pineal organ directly receives light stimuli and secretes melatonin under darkness. Once released into blood, melatonin acts on the hypothalamo–hypophyseal–gonadal axis to regulate oocyte development from primary oocytes to mature oocytes. Melatonin may directly act on oocytes to interact with other hormones, such as maturation-inducing hormone (MIH) and serotonin (SER), to induce final maturation by active formation of maturation-promoting factor (MPF) – a complex of cyclin B and cyclin-dependent kinase (Cdk1). Melatonin injection or photoperiodic manipulation may lead to an acceleration of the process of transformation of stage-I to stage-II oocytes (progonadal) during the preparatory phase, or retardation of oocyte growth (antigonadal) in the pre-spawning and spawning phases, or no subtle changes in the ovary during the post-spawning phase. Ach, acetyl choline; cGV, central germinal vesicle; DA, dopamine; 17-βE_2_, estradiol; GnIH, gonadotropin-inhibitory hormone; GnRH, gonadotropin-releasing hormone; GtH, gonadotropic hormone; GVBD, germinal vesicle breakdown; MEL, melatonin; MT-R, melatonin receptors; NE, norepinephrine; Pt, pituitary gland; Vg, vitellogenin.

Earlier mammalian studies documented clearly that the effects of exogenous melatonin administration on the reproductive functions are dependent on the dose, duration, and time of treatment (26). Accordingly, influences of daily evening injection of melatonin on the activity of gonads were examined in relation to the dose (25, 50, or 100 μg/100 g body weight/day) of hormone, duration (1, 15, or 30 days) of treatment, and the reproductive status of the fish. Due to lack of any prior data, the employed doses of melatonin were relatively higher than the physiological levels. Unlike any other fish study, effects of an identical dosage regimen of melatonin administration on the functions of ovary (23) and testis (27) were studied in *C. catla* during the four different reproductive (*viz*., the preparatory, the pre-spawning, the spawning, and the post-spawning) phases of an annual cycle (Figure 1). In such study, single administration of melatonin, irrespective of its dose, could not alter the functional status of gonads in any part of the annual cycle. However, daily melatonin treatment, at each employed dose, over a period of 15 or 30 days resulted in an inhibition in the ovarian activities in a duration (15 days < 30 days)-dependent manner during pre-spawning and spawning phases of the reproductive cycle. Melatonin treatment for 50 or 60 days suppressed the development of gonads in *Oryzias latipes*, *Fundulus similis*, and *Heteropneustes fossilis* [for references, see Ref. (1)], whereas in carp, antigonadal effects of melatonin were noted even after a shorter duration (15 days) of treatment during pre-spawning as well as spawning phases of reproductive cycle. Further demonstration of a dose-dependent (25 < 50 < 100 μg) inhibitory effects of melatonin treatment on the functions of carp gonads lend support to the conjecture that gonadal response to exogenous melatonin in a particular reproductive phase would essentially depend on the dose as well as duration of hormonal administration (23).

One of the unique features of carp study was that prolonged (30 days) treatment of melatonin at relatively higher doses (50 or 100 μg/100 g body weight) resulted in precocious growth of ovaries and testes to indicate a progonadal response during the preparatory phase while no subtle changes in gonads during the post-spawning phase. Earlier studies in few teleosts also reported pro- or antigonadal influences of melatonin under varied experimental conditions. Melatonin administration in high dose led to an antigonadal response in both sexes of *Gasterosteus aculeatus* under 16L:8D, but in its low dose induced progonadal effects under LP (28). In *Clarias batrachus*, testosterone levels during the pre-spawning phase decreased after melatonin injection at the dosage of 100 or 200 μg/fish, but elevated at the dose of 400 μg/fish (29), indicating that higher dose of melatonin could induce counter-inhibitory effects. The carp study depicted that gonadal response to melatonin treatment in both females (23) and males (27) vary with the dose and duration of treatment as well as the sexual status of fish. A recent study on *Clarias macrocephalus* reported that exogenous melatonin (50 mg/kg in the diet) feeding administration for 8 weeks could significantly improve the first puberty event by enhancing the maturation of testes and sperm (30).

### The Actions of Melatonin on the Steroid-Induced Maturation of Fish Oocytes

Fish oocyte maturation is a naturally occurring process in which two successive meiotic divisions take place producing two polar bodies, when the enlarged nucleus of the primary oocyte, which is arrested at the diplotene stage, shifts toward a more peripheral position, followed by the breakdown of its membrane and with the extrusion of a polar body that ultimately mark completion of the first meiotic division (31). At the transition of prophase/metaphase, oocyte nuclear envelope denatures or germinal vesicle breakdown (GVBD) occurs to indicate completion of the penultimate step in the progression of oocyte maturation (32). Second meiotic division starts immediately thereafter, but progression continues only up to the metaphase stage during which a distinct animal pole differentiates from the vegetal pole followed by yolk maturation and hydration resulting in less opaqueness. The ovulated eggs are spawned in water and are fertilized by the male immediately after the mature oocyte is ovulated out of its follicular envelope.

In vertebrates, pre-ovulatory gonadotropin surge stimulates final oocyte maturation or resumption of meiosis. The promoting effects of gonadotropins on the oocytes of most fish species involve two major mechanisms in two temporally different developmental stages (33): (a) a steroid-independent priming stage that increases the maturational competence of the oocytes and (b) a maturational stage that depends on the gonadotropin stimulated synthesis of a steroid – 17α, 20β-dihydroxy-4-pregnen-3-one (17α, 20β-DHP), commonly known as maturation-inducing steroid (MIS) or maturation-inducing hormone (MIH), involving steroidogenic pathways in the ovarian follicular cells (33). In fish, final step of oocyte maturation is initiated by the potent MIH that induces formation of maturation-promoting factor (MPF) in the oocyte cytoplasm by acting on the membrane of oocytes. MPF comprises two subunits: (i) cyclin B, a regulatory subunit and (ii) cyclin-dependent kinase (Cdk1, Cdc2, or p34 kinase), a catalytic subunit of which the activation of cdc2 is related with its mobility shift from 35 to 34 kDa (34).

Although several hormones are known to act as inducer of MPF synthesis (32), influences of any indolamine on the steroid-induced maturation of oocytes and/or formation of MPF in fish oocytes were studied for the first time in carp (35). The *in vitro* study revealed that melatonin accelerates the action of MIH when added 4 h prior to MIH in the incubation medium (Figure 1). Densitometric analysis of the immunoblot data collected from the homogenates of melatonin pretreated MIH incubated oocytes showed that cyclin B level continued to increase even after 4 h of incubation and reached its peak after 12 h. Moreover, determination of H1 kinase activity as an indicator of MPF activity in oocytes provided indication that melatonin pre-incubation increased MIH stimulation of histone H1 phosphorylation as compared to MIH alone. This study presented the first evidence of extra-hypothalamic action of melatonin in the process of oocyte maturation in any fish species (35). The hypothesis earned further support from another *in vitro* study on zebrafish (*Danio rerio*), in which melatonin exerted a direct action on follicles (as shown by the increase of the oocytes undergoing to GVBD) and modulated *mpr* α and β gene (whose proteins are involved in oocyte maturation) expression (36). Further study of the actions of serotonin (the precursor of melatonin) on oocyte maturation in carp indicated that incubation of oocytes with serotonin, or melatonin, or both serotonin and melatonin diminished the stimulatory actions of melatonin and MIH on the rate of GVBD (37).

## Mechanism of Actions of Melatonin as a Hormone in the Regulation of Fish Reproduction

The mechanism by which melatonin regulates fish reproduction is one of the major areas that received serious attention in the studies on different fish, but is not yet clearly understood. Possibly, melatonin interacts with the brain–pituitary–gonad axis, or with one or more of a variety of peripheral and/or central sites, including the brain (38), the pituitary (39), and the gonads (35, 40). It seems likely that melatonin acts on the HPG axis and on the ovary itself (36, 38, 40).

### Actions of Melatonin on the Hypothalamo–Hypophyseal–Gonadal Axis

The hypothesis emerged from fish studies in common states that melatonin actions on the reproductive system result from an interaction with the hypothalamic control of pituitary functions. In periodic breeders, where melatonin provides temporal information related to day length and seasons, the hypothalamo–hypophyseal system plays a major role in controlling reproduction (39). However, pathways of hypothalamus-mediated melatonin actions in animals may vary with the organization of their hypothalamic neurosecretory system. In fish, the preoptic area (POA) of hypothalamus receives nervous inputs from the retina and the pineal organ to integrate photoperiodic information from various sources, but the hormonal (melatonin) input from the pineal organ occupies a pivotal position in the photoneuroendocrine control of fish reproduction (4). Neurons from the POA transmit monoaminergic (i.e., dopamine, 5-hydroxytryptamine) or peptidergic signals to the pituitary, corresponding to peptides (e.g., isotocin and arginine vasotocin) of the neurohypophysis or releasing/inhibiting hormones acting on different adenohypophyeal cells to regulate synthesis and release of different hormones associated with seasonal growth of gonads. Moreover, detection of melatonin receptors (both MT1 and MT2 subtypes) and display of binding of ^125^IMel to membrane preparations in pike and trout pituitary glands provide evidence that melatonin might modulate neuroendocrine functions by targeting the pituitary gland itself (41). It seems likely that varied influences of melatonin in different parts of the reproductive cycle depend on the type of melatonin receptors expression in the pituitary glands (39). Obviously, melatonin *per se* is neither antigonadotrophic nor progonadotrophic rather the changing duration of nocturnal melatonin may act as a passive signal that provides the HPG axis information as to the time of year (12, 42). The reproductive axis uses the season-related melatonin rhythms to adjust timing of reproduction accordingly. Since the hypothalamus is the highest regulatory center of pituitary functions, several studies emphasized the mechanism of melatonin actions on this part of brain.

#### Hypothalamic Actions of Melatonin – Possible Role of Gonadotropin-Releasing Hormone and Gonadotropin-Inhibitory Hormone

Gonadotropin-releasing hormone (GnRH) is a decapeptide, which is synthesized and released by hypothalamic neurosecretory cells in a pulsatile pattern into the hypothalamo–hypophyseal portal circulation, and plays a central role in the regulation of reproduction. This peptide binds and activates its cognate receptor (GnRH receptor) on the pituitary gonadotrope cells and, in turn, stimulates the synthesis and secretion of gonadotropins (GtHs). Out of so far identified 30 structurally different forms of GnRH, 18 structural variants of GnRH are found in vertebrates (43). Several studies provided evidence in favor of expression of three different GnRH forms, i.e., GnRH 1, GnRH II, and GnRH III, among which GnRH III has complete sequence conservation and is present only in teleosts (44). The GnRH receptor belongs to the rhodopsin-like G-protein-coupled receptor (GPCR) superfamily, which contains a characteristic seven transmembrane (TM) domain structure (45). Existence of day–night variations and melatonin inhibitory effects on the expression of GnRH 1, GnRH 3, and respective GnRH receptors in the brain are known for European sea bass (*Dicentrarchus labrax*) (46). Obviously, possible interactions between melatoninergic and GnRH systems could represent a substrate of photoperiod effects on reproductive and other physiological events in different fish species, in which melatonin is directly implicated to photoperiodic regulation of seasonal reproduction.

The recent discovery of a novel hypothalamic gonadotropin-inhibitory hormone (GnIH) in several vertebrates, including fish, added a new dimension to the present understanding on the mechanism of hypothalamic control of reproduction (47–49). GnIH, *via* binding to GnIH receptor (GnIHR), plays a negative role on the avian and mammalian reproductive axis by inhibiting luteinizing hormone (LH) release. In birds, the GnIH receptor, comprising seven TM domains that specifically bind to GnIH, expresses in the pituitary and several brain regions including the hypothalamus (50). Although information are scanty on the GnIH/GnIHR system in lower vertebrates, the orthologous gnih genes in stickleback, medaka, and Takifugu, and three orthologous genes (gnihr1, gnihr2, and gnihr3) for the gnihr were identified in zebrafish (51). These three zebrafish gnihrs are typical seven TM GPCRs sharing high sequence homology with the mammalian and avian GnIHRs (GPR147). Earlier studies showed that goldfish gnih peptide could actually stimulate LH and FSH release from cultured salmon pituitary cells (52). In contrast, further studies on mature female goldfish revealed that GnIH orthologs inhibit gonadotropin release (51, 53), indicating a conserved role for GnIH and its orthologs in the control of the HPG axis across species. Nonetheless, the function of fish GnIH orthologs remains inconclusive because the physiological properties of fish GnIH peptides are debatable (54).

Recent findings provide evidence that melatonin induces GnIH expression in GnIH neurons and thereby influences synthesis and release of GnIH from hypothalamic neurons, but existing information is limited only to photoperiodic birds and mammals; moreover, the actions of melatonin on GnIH expression across species are not identical (49). Melatonin seems to act directly on GnIH neurons to stimulate expression and release of GnIH stimulates GnIH expression in quail, a photoperiodic bird (55, 56). A similar, but opposite, action of melatonin on the inhibition of the expression of GnIH is found in photoperiodic mammals, such as Syrian and Siberian hamsters and sheep (48). Although discrepancy between these studies remains unexplained, it appears possible that in quail melatonin through its receptors (Mel1c) triggers different intracellular signals in GnIH neurons, but in photoperiodic mammals, melatonin indirectly regulates GnIH expression due to absence of Mel1c melatonin receptor subtype in GnIH neurons (48). However, despite demonstration of the stimulatory effects of melatonin on GnIH mRNA expression in brain cells of goldfish (*Carassius auratus*) (57), the mechanism of melatonin actions on GnIH neurons in controlling seasonal reproduction in teleosts remains unknown and thereby warrants further study.

#### Kisspeptin in Concert to Melatonin Synchronized Reproduction

The identification of kisspeptins (KiSS) as new hypothalamic peptides and their GPCR GPR54 is a major advance in understanding the neuronal mechanisms controlling GnRH secretion and, thus, gonadal functions in fish (58). The kisspeptins are a family of neuropeptides which act as upstream stimulators of GnRH neurons (59). More specifically, kisspeptins appear to play an important role in determining seasonality of reproduction, transducing the feedback effect of gonadal steroids as well as having an independent (non-steroid dependent) circannual rhythm (58, 60–62). The KiSS genes have been identified in most vertebrates and fish, which may have up to three Kiss genes (63). The genes that are emerging as important regulators of GnRH and the reproductive axis, *kiss1* (encoding Kisspeptin-1), *kiss2*, and their G-protein-coupled Kisspeptin receptors (kissr1 and kissr2) have been cloned in the teleost (58, 64, 65). In zebrafish (*D. rerio*), medaka (*O. latipes*), and goldfish (*C. auratus*), two genes encoding Kissr have been identified (66), whereas only *kiss2* has been identified in several fish species such as puffer fishes [green-spotted puffer (*Tetraodon nigroviridis*), torafugu (*Takifugu rubripes*), and three-spined stickleback (*G. aculeatus*)] (58). Despite demonstration of the ability of both Kiss1- and Kiss2-encoded Kp-10 to activate the gonadotropic axis in zebrafish and sea bass, the Kiss2 decapeptide is pointed to be apparently more effective in terms of activation of pituitary gonadotropin gene expression and secretion (61). Further functional studies in goldfish provide evidence that the Kiss1-encoded decapeptide in this species is a more potent gonadotropin secretagogue (67). Available data, therefore, suggest important species differences regarding which Kiss system is actually the dominant in the control of the HPG axis in fish (58). In medaka (*O. latipes*), *kiss1* and *kiss2* are expressed in distinctive hypothalamic neuron populations (60). The regulation of reproduction by the hypothalamic Kisspeptin–Kiss receptor–GnRH system in fish (Figure 1) is supposed to perform functions *via* melatonin signaling by both directly regulating KiSS-1 expression and changing sensitivity of KiSS-1 to sex steroid feedback (68). In sea bass, kiss1- and kiss2-immunoreactive neurons are identified in the lateral tuberal nucleus and the parvocellular preoptic nucleus, respectively (69), and two cell masses also express melatonin receptors (70). Since kisspeptins are associated with the onset of puberty in Nile tilapia (22), it has similar GnRH regulatory abilities (71). The results of zebrafish study by showing a receptor-mediated action of melatonin at the level of brain indicate that melatonin, probably involving the Kiss/Gpr54 system in the GnRH neurons, may stimulate the release of hypothalamic GnRH (36). The hypothesis earns further support from the analysis of transcriptional activity of gonadotropin-releasing hormone (gnrh), luteinizing hormone receptor (lhr), and melatonin receptor (mtnr) in the female killifish brain (40). Despite demonstration of a major role of melatonin in the transduction mechanism of photoperiodic signals in the control of seasonal reproduction, kisspeptin cells do not appear to express the melatonin receptor, so the means by which seasonality changes the activity of kisspeptin remains unknown (59). However, a recent study on goldfish provided indications that light-mediated actions of melatonin to control sexual maturation in fish might be the outcome of interactions between melatonin, GnIH, and Kiss (57). Thus, functional interplay between the GnRH, GnIH, kisspeptin, and melatonin at the level of hypothalamus in the regulation of reproduction in seasonal breeders remains as an important area of future research.

#### Demonstration of Melatonin Receptors in Carp Oocytes – An Evidence of Direct Hormonal Action of Melatonin on Fish Gonads

Demonstration of melatonin receptors in isolated human oocytes (72) and in rat ovaries (73) supported the notion of a direct hormonal effect of melatonin on ovarian functions in mammals. However, no data on melatonin receptors on the gonads of any fish species were available till use of an anti-MT1 goat polyclonal antibody in Western blot analysis detected a band of 37-kDa corresponding to Mel_1a_ melatonin receptor (Mel_1a_R) proteins in the carp ovarian homogenate (74). This study also provides first information on the localization of Mel_1a_R melatonin receptor proteins in different cellular fractions of ovary and their diurnal profiles in different parts of an annual reproductive cycle to focus its temporal relation with the serum melatonin titers (17, 74). Mel_1a_ melatonin receptor proteins are present in both the membrane and cytosolic fractions of the carp ovarian homogenate, though the relative band intensity (a ratio of the band intensity of Mel_1a_R to β-actin) of ovarian Mel_1a_R is always greater in the membrane fraction than in its cytosolic counterpart. Functional role of membrane-bound Mel_1a_R in the mediation of intracellular effects of melatonin is well known (75, 76), but the significance of cytosolic Mel_1a_R remains obscure. In a diurnal cycle, immunoreactivity of Mel_1a_R protein in the carp ovary is highest at midnight and lowest at midday. The pattern of day–night rhythms in ovarian Mel_1a_R does not vary in relation to the reproductive status of the carp or changes in photo-thermal conditions of the environment in an annual cycle. However, the nocturnal expression of ovarian Mel_1a_R varies in relation to the reproductive stages in an annual cycle, as it reaches peak during the spawning phase and becomes lowest during the post-spawning phase. The profiles of serum melatonin and Mel_1a_R in the ovary of carp exhibit an identical pattern of diurnal variations in each reproductive phase, but not in the preparatory phase when diurnal peak values of serum melatonin (in late dark phase) and of ovarian Mel_1a_R (at midnight) are completely dissociated from each other (77). Such findings may point to an unexplained mechanism of action of melatonin on carp gonads. For example, daily injection of melatonin at 25 μg/100 g body wt for 30 days in male (27) and female (23) carp leads to a progonadal response during the preparatory phase, but antigonadal or no response in the remaining parts of the reproductive cycle (Figure 1). Thus, the findings argue that reproductive phase-dependent effects of exogenous melatonin on gonads in carp may be due to the respective season-related diurnal levels of endogenous melatonin and/or its interactions with other hormones, rather than to the profiles of ovarian melatonin receptors (77). Nonetheless, supporting data from carefully controlled further experimental studies would be required to justify this hypothesis. The *in vitro* modeling might solve one of the central questions of melatonin physiology: how the rhythmic patterns of melatonin and its receptors on the ovary are associated with the photoperiodic response of concerned fish. The location of the melatonin target for photoperiodic regulations is not yet determined quite precisely, but available data may argue that melatonin might act through more than one structure even within one species (76). In essence, the findings on the localization and temporal pattern of melatonin receptors on the carp oocytes offer interesting perspectives to explain the control mechanisms of its rhythms and response to external cues such as photoperiods and other hormones. Moreover, the results obtained in carp (Figure 1) might permit insights into the physiology of melatonin in the regulation of fish reproduction.

## Actions of Melatonin as Antioxidant in the Regulation of Fish Reproduction

Generation of a large amount of free radicals during oocyte maturation and ovulation is known to cause an elevated oxidative stress (78). Follicle development in the mammalian ovary is a complex physiological process during which active synthesis of steroids is accompanied by production of large amount of free radicals, especially reactive nitrogen species (RNS) and reactive oxygen species (ROS) (79, 80). Excessive amount of free radicals, especially ROS, leads to oxidative stress that in turn causes damage of oocytes, accelerates oocyte aging, and deteriorates oocyte quality (81). Melatonin, by playing the role of a free radical scavenger (82), may minimize the free radical damage in the ovary and ultimately improve the quality of oocytes (83, 84). *In vitro* study on isolated oocytes reveals that elevation of melatonin level in the ovary depends upon follicular growth during the complex process of ovulation (85). Data are also available to suggest that melatonin may act on various cells of ovarian follicles as an indirect antioxidant to activate major antioxidative enzymes, such as superoxide dismutase (SOD), catalase (CAT), and glutathione peroxidase (GPx), which metabolize free radicals to reduce oxidative stress *in vivo* (12). SOD together with GPx and CAT form the main enzyme defense mechanism against harmful effects of free radicals. Glutathione *S*-transferase (GST) is also an important enzyme in the glutathione redox cycle. Both GST and reduced glutathione (GSH), a non-enzymatic antioxidant, participate in the removal of toxic free radicals. Thus, it is evident that melatonin, by playing the role of an antioxidant, may minimize the free radical damage in the ovary, ultimately leading to improve the quality of oocytes (83, 84). However, until recently, the hypothesis emerged from mammalian studies was not tested in any fish species in which poor egg quality is a major constrain for induced breeding.

**Figure 2:**
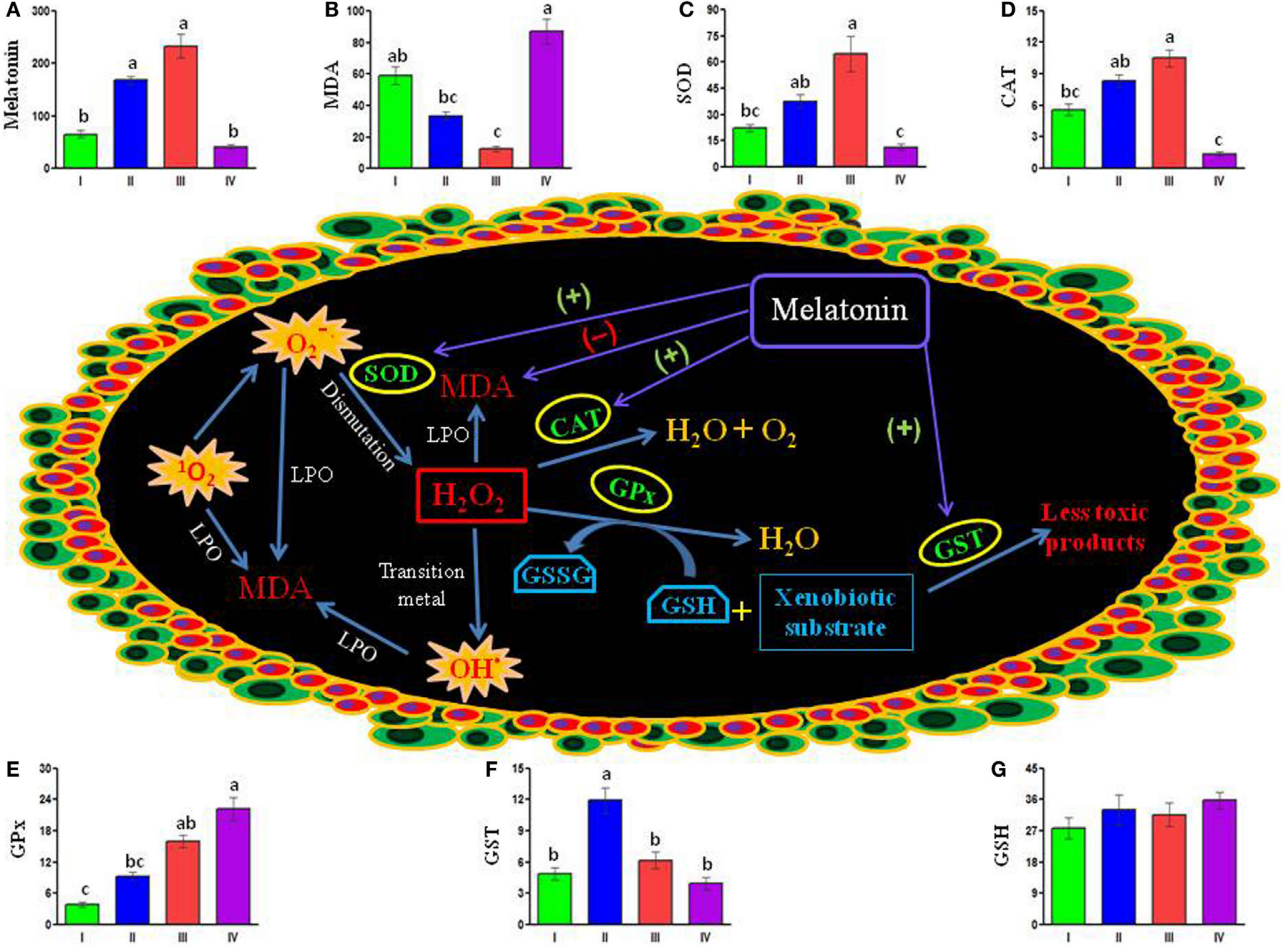
**Schematically presented available data depict possible interactions between melatonin, antioxidant enzymes, and stress-inducing free radicals [^1^O_2_, ${\text{O}}_{2}^{•-}$, and OH (marked as explosion)] in the carp oocytes**. Ovarian melatonin may stimulate (+) the activity of superoxide dismutase (SOD), catalase (CAT), and glutathione-S-transferase (GST), but reduce (−) the level of malondialdehyde (MDA) – a faithful marker of intracellular stress. Notably, ovarian melatonin titers do not show any significant correlation with the activity of glutathione peroxidase (GPx) or the level of GSH (reduced glutathione). Histogram represents mean values of **(A)** melatonin (picogram per gram tissue), **(B)** stress marker MDA (nanomole per milligram protein), different enzymatic antioxidants (unit per milligram protein) **(C)** SOD, **(D)** CAT, **(E)** GPx, **(F)** GST and non-enzymatic antioxidant, and **(G)** GSH (nanomole per milligram protein) in the carp ovary during preparatory (I), pre-spawning (II), spawning (III), and post-spawning (IV) phases of an annual reproductive cycle. GSSG, oxidized glutathione; H_2_O_2_, hydrogen peroxide; LPO, lipid peroxidation; ^1^O_2_, singlet oxygen; ${\text{O}}_{2}^{•-}$, superoxide anion; OH^·^, hydroxyl radical [cf., Ref. (86)].

A recent study on carp ovary under natural photothermal conditions provides the first information on the profiles of melatonin, malondialdehyde (MDA), a specific marker of intracellular oxidative stress, and enzymatic as well as non-enzymatic antioxidants, in relation to the dynamics of oocyte development during the four distinct reproductive phases in an annual cycle (86). Significant seasonal variations in MDA levels with a peak in the post-spawning phase and gradual decrease thereafter toward seasonal minimum value during the spawning phase denotes ovarian oxidative stress as very high during the post-spawning season and low during the spawning phase of annual cycle. A significant negative correlation between the levels of melatonin and MDA in the ovary points to a possible role of ovarian melatonin as an antioxidant. The study also reveals that the activity of SOD, CAT, and GPx in the ovary varies significantly in between different reproductive phases in an annual cycle. However, the temporal pattern of their activity in an annual cycle does not seem to be identical. While the activity of both SOD and CAT follows similar pattern of seasonal variations with a peak during the spawning, seasonal maximum values of GST and GPx are noted during the pre-spawning and the post-spawning phases, respectively. The levels of reduced glutathione (GSH), unlike the activity of studied antioxidant enzymes, in the ovary do not show any significant variations in between different sampling months in an annual reproductive cycle. Moreover, correlation coefficient analysis of data indicating a positive correlation between the seasonal values of melatonin and the activity of SOD, CAT, and GST in the ovary underlines their functional interplay as well as importance of ovarian melatonin in reducing oxidative stress (Figure 2). Taken together, obtained data are consistent with the findings on human granulosa cells where a direct stimulatory effect of melatonin on the activity of antioxidative enzymes, such as SOD and CAT, is suggested to improve the quality of oocytes (78, 81, 87). Thus, the data presented in carp study (86) suggest that physiological actions of melatonin in reducing oxidative stress in the ovary may be related to the activity of antioxidant enzymes, such as SOD, CAT, and GPx (88), but not to the level of non-enzymatic antioxidant (GSH). It is possible that ovarian melatonin acts at more than one points in the antioxidant defense system (Figure 2), rather than on the activity of a particular antioxidative enzyme/agent. Obviously, the mechanism of actions of melatonin as an antioxidant on the carp oocytes remains speculative. Nonetheless, in the current state of knowledge, it appears reasonable to argue that ovarian melatonin is one of the physiological variables that may reduce oxidative stress during seasonal growth and development of ovary and maturation of oocytes.

Considering limitations of the study under natural conditions (86), it appears necessary to document experimentally that the fish injected with GnRH and/or gonadotropic hormones to stimulate ovarian growth exhibits the profiles of ovarian melatonin and different antioxidants corresponding to those noted during spawning phase under natural conditions. Thus, in a very recent study, the process of final oocyte maturation and oxidative status of the pre-ovulatory follicles in the ovary of gravid carp is studied at different time intervals following injection with melatonin and/or ovaprim (synthetic GnRH and domperidone), or luzindole (a pharmacological blocker of melatonin receptors) before them (89). It is notable that melatonin treatment 2 h before ovaprim injection causes shortest latency period and highest rate of ovulation due to formation of MPF. Exogenous melatonin, irrespective of injection schedule, leads to significant reduction in intrafollicular oxidative stress and an increase in the ovarian levels of both enzymatic and non-enzymatic antioxidants, melatonin, and its receptor proteins. Ovarian melatonin concentrations show a significant negative correlation with the level of oxidative stress, but a positive correlation with the rate of GVBD and the activity/level of different antioxidants as well. However, melatonin and/or ovaprim do not evoke any response in luzindole pretreated carp. Thus, the study provides clear indications that melatonin pretreatment in carp ameliorates ovaprim actions on the process of final oocyte maturation by formation of MPF and alleviates oxidative stress in the pre-ovulatory follicles by stimulating different antioxidants (89). However, demonstration of individual role of different antioxidant agents in mediating melatonin actions in the process of final oocyte maturation in carp and other fish species remains as an exciting area of future research.

## Conclusion

The information already gathered from the studies on different of temperate fish species and subsequently on subtropical carp provides convincing data to suggest that melatonin plays an important role in the regulation of cascade of reproductive events in an annual cycle. Physiological significance of melatonin synthesized in the pineal organ in synchronization with environmental light–dark cycles has so far focused mostly on its role of an endogenous signal of darkness in a changing environment. Specific receptor-mediated actions of melatonin on the target cells are now evident at various levels of regulatory axis of gonadal gametogenic and steroidogenic functions. It is generally agreed that melatonin as a hormone acts on the hypothalamo–hypophyseal system or directly on the gonads or at both the levels. This tryptophan derivative seems to play a critical role in controlling the endocrine axis starting from the kisspeptin that in turn influences hypothalamic neurons to produce GnRH and/or GnIH to regulate gonadotropic functions of the adenohypophysis. On the other hand, melatonin by acting directly on gonads may regulate transcription of genes whose proteins are involved in the synthesis of gonadal steroids to control oocyte competence and maturation. Additionally, demonstration of unique action of melatonin as a potent scavenger of free radicals or an antioxidant has opened up further possibility that this tiny indole substance by acting locally may reduce oxidative stress of the growing ovarian follicles may play a physiological role in the regulation of seasonal reproduction by inducing oocyte maturation during the act of spawning. Though melatonin detected and measured in the carp ovary exhibits seasonal variations, it remains unclear whether measured melatonin is endogenously synthesized or taken up from the circulation. Moreover, information base on the antioxidant actions of melatonin in the regulatory process of oocyte maturation in fish is so far limited only to the carp study and, thus, warrants further investigation on other fish species. An optimistic view suggests that future carefully controlled studies on more variety of fish species would potentially contribute to current understanding on the mechanisms by which this lipophilic bio-molecule regulates the oxidative status of oocytes during their conserved seasonal breeding cascade. It would also be imperative to ascertain whether melatonin acts as an antioxidant at every levels of the regulatory axis of seasonal reproduction, and melatonin-induced reduction of oxidative stress of the oocytes is a prerequisite for its meiotic resumption in an annual reproductive cycle.

## Author Contributions

Both the authors contributed equally in preparation of the manuscript.

## Conflict of Interest Statement

The authors declare that the research was conducted in the absence of any commercial or financial relationships that could be construed as a potential conflict of interest.
